# Commentary: Oil degradation and biosurfactant production by the deep sea bacterium *Dietzia maris* As-13-3

**DOI:** 10.3389/fmicb.2015.01557

**Published:** 2016-01-12

**Authors:** Pattanathu K. S. M. Rahman, Kamaljeet K. Sekhon Randhawa

**Affiliations:** ^1^Technology Futures Institute, School of Science and Engineering, Teesside UniversityMiddlesbrough, UK; ^2^Section for Sustainable Biotechnology, Aalborg UniversityCopenhagen, Denmark

**Keywords:** genus *Dietzia*, di-rhamnolipids, biosurfactant, pathogenesis, alkanes, biosynthetic pathway

Surfactants or surface active agents find broad industrial applications as additives—in household detergents, cleaning products, toothpastes, paints, coatings, leather, paper, cosmetics, pharmaceuticals, food products, and agriculture. Irrespective of their interesting applications, all surfactants end up their journey in sewers or drains—from where they directly or indirectly affect the flora, fauna, and water bodies causing environmental concerns.

Biosurfactants—plant based or produced by microbes are considered the holy grail of “natural” products. The versatility of biosurfactants is due to their amphiphilic property—a sought-after attribute—which gives them a double affinity for both hydrophilic and lipophilic compounds. Biosurfactants are therefore “detergents” which help lower the surface tension of a liquid or interfacial tension between two liquids. Their eco-friendly origin makes them stand tall among their synthetic or petroleum based counterparts. Regardless of the technology that has been employed to produce biosurfactants their sky-high prices are still a reason of concern for the biosurfactant research community anticipating more intensive research. Apart from the innovative production technologies tried so far, there are a number of microbial strains which have also been harnessed in these technologies for cost-effective generation of biosurfactants.

A detailed and effective study is conducted by Wang et al. (2014) showing the complete pathway of the di-rhamnolipid synthesis process in the genus *Dietzia* isolated from extreme environments. This short account will present an annotation on the highlights of the study with some critical analysis. In this study the authors have explored the possibility of biosurfactant production by *Dietzia maris* As-13-3, which was originally isolated from deep sea hydrothermal field environment of Southwest Indian Ocean (Chen and Shao, 2009). Grown on 2% (v/v) n-hexadecane as the sole carbon source this deep sea bacterium was shown to produce di-rhamnolipid as biosurfactant. Analysing *Dietzia maris* As-13-3 for various activities, the authors claim that the strain could play significant role in oil degradation, biosurfactant production, and marine oil removal.

Rhamnolipids are the most studied biosurfactants and there are now companies entirely dedicated to their research and production such as AGAE Technologies Ltd., Jeneil Biosurfactants, and Paradigm Biomedical Inc., all based in USA (Sekhon et al., 2012). Nevertheless, there is enormous scope for investigating new and extreme environments so as to look for novel strains which can mass produce these biosurfactants using relatively cheap substrates. Microbial consortia ERCPPI-2 isolated from heavy crude oil contaminated site in Iran, *Virgibacillus salarius* isolated from soil and sea water samples from Saudi Arabia, 10 thermohalophilic isolates of genus *Bacillus* isolated from Iranian petroleum reservoirs are few examples of extremophilic microorganisms that produce biosurfactants (Darvishi et al., 2011; Zargari et al., 2014; Elazzazy et al., 2015). Although alkanes are considerably not very cheap but interestingly *Dietzia maris* As-13-3 could utilize three different alkanes such as n-tetradecane, n-hexadecane, and pristane, for biosurfactant production. The strain could utilize short-chain and middle-chain n-alkanes from C_8_ to C_20_, and vigorous degradation was reported for C_14_ to C_18_ and pristane (Wang et al., 2014). It is also quite fascinating how the authors summarized that n-hexadecane serves as the main raw material for production of biosurfactant by this strain and revealed a complete pathway for the biodegradation of alkanes by terminal oxidation—mentioning the genes including *alkB, cyp153, algC, rmlA, rmlB, rmlC, rmlD, rhlA, rhlB*, and *rhlC* which are categorically induced by alkanes for biosurfactant production.

The rhamnolipids produced by *Dietzia maris* As-13-3 at a CMC of 120 mgL^−1^ lowered the surface tension of water from 74 ± 0.2 to 38 ± 0.2 mN m^−1^ (Wang et al., 2014), which was not as good as 29 mN m^−1^ with a CMC value in range of 5-60 mgL^−1^ by *Pseudomonas aeruginosa* (Van Dyke et al., 1993) but better than 42 mN m^−1^ at a CMC value of 225 mgL^−1^ by *Burkholderia thailandensis* (Dubeau et al., 2009). The comparison with surface tension values of rhamnolipids produced by other significant strains gives a very clear picture of the effectiveness and efficacy of these biosurfactants—efficient biosurfactants have a low CMC value. Future studies in the field would, however, have benefitted from a comparative analysis of the total yield of rhamnolipids produced per liter of the media by this deep sea bacterium and throwing light on the prospective commercialization of these rhamnolipids.

Genus *Dietzia* is little unknown but contains clinically significant organisms. This emerging actinomycetes has been misidentified as *Rhodococcus equi* to which it has remarkable similarities—for instance other species of this genus including *Dietzia maris* showed 98.1% similarity to *Rhodococcus* sp. (Koerner et al., 2009). *Dietzia maris* taxon, initially proposed for two strains isolated from flatfish halibut, was classified as “*F.maris*” (Harrison, 1929). Surprisingly, there are three reports of *Dietzia maris* causing infections in humans (Koerner et al., 2009) and has been clearly implicated as potential opportunistic human pathogen (Bemer-Melchior et al., 1999; Pidoux et al., 2001; Reyes et al., 2006). Having said that, to date there has been no attempts to deeply investigate the pathogenicity factors of *Dietzia maris*—which apparently colonizes human skin as it excretes n-alkanes (Koerner et al., 2009). In view of these studies it will be too early and hasty to say and claim that *Dietzia maris* As-13-3 is a “non-pathogenic” strain and the rhamnolipids are suitable for commercial production and are industrially safe specially in the food and pharmaceutical sector—without thoroughly inspecting the virulence factors of this deep sea bacterium.

This study has certainly outdone several other studies in terms of the extensive new insights into the genetics and physiology of the genus *Dietzia*—however a few more significant tests could have really elaborated how efficient and successful this deep sea bacterium *Dietzia maris* As-13-3 is in the production and commercialization of di-rhamnolipids. Such studies undoubtedly bring into forefront the less explored genus, thereby increasing our knowledge in terms of the amount of work that still can be undertaken for search of efficacious strains from extreme and unexploited environments.

## Author contributions

PR initiated the research topic and co-ordinated the entire editorial process. There are 11 manuscripts accepted for publication in this research topic contributed by 55 authors from UK, Denmark, Greece, Germany, South Africa, India, Brazil, Bahrain, Portugal, and China. He has initiated peer review process by inviting experts from Germany, Spain, USA, Trinidad and Tobago, India, Denmark, China, Bahrain, Malaysia, Japan, and UK. The reviewers co-operation and timely responses to complete the research topic is highly commendable. One of the articles, submitted for the research topic by Wang et al. (2014), Oil degradation and biosurfactant production by the deep sea bacterium *Dietzia maris* As-13-3 is selected for General commentary. KS has contributed as Co-author in this Editorial commentary. She is an expert on this topic and has been working specifically in this field, she has her adept inputs in conceptualizing the commentary, analysing the data, interpreting and drafting the commentary. KS has given her final approval of the version to be published and agrees to be held accountable for all aspects of the work related to the accuracy and integrity.

### Conflict of interest statement

The authors declare that the research was conducted in the absence of any commercial or financial relationships that could be construed as a potential conflict of interest.
